# Arnie Levine and the MDM2–p53 discovery: a postdoctoral fellow’s perspective

**DOI:** 10.1093/jmcb/mjz073

**Published:** 2019-08-20

**Authors:** Gerard P Zambetti

**Affiliations:** Department of Pathology, St. Jude Children’s Research Hospital, 262 Danny Thomas Place, Memphis, TN 38105, USA

It was early 1990 that we had gathered at Arnie Levine’s home for dinner to celebrate the transition of several postdoctoral fellows to faculty positions in academia. As a new postdoctoral fellow in his lab, I was thrilled to have the opportunity to spend some personal time with Arnie. We were discussing the paradigm shift made by his group a year earlier that p53 is a tumour suppressor (Finlay et al., 1989) and not an oncogene, as originally thought for a decade after its original discovery (Lane and Crawford, 1979; Linzer and Levine, 1979). Paraphrasing Arnie’s comment about this landmark discovery, he said that it was fun, a really big wave to ride, and not to worry as there will be more to come. Unknowingly at the time, that prediction led to the identification of Mdm2 as a negative regulator of p53. This perspective revisits some personal experiences of training as a postdoctoral fellow under the mentorship of Dr Arnie Levine and highlights several key experiments that established Mdm2 as the negative regulator of p53.

## Graduate student education and training—how do you pick a mentor?

I must say that I have been extremely fortunate to have been trained by several outstanding mentors. Synonyms of ‘mentor’ based on the Merriam-Webster Thesaurus include coach, counsel, guide, and pilot. A good mentor is someone who is committed to their trainees and is invested in their success. I joined the lab of Drs Gary and Janet Stein as a graduate student at the University of Florida. Shortly after becoming a member of their group, they advised me to carry out my postdoctoral studies with an esteemed colleague of theirs in the Northeast. They even arranged for me to visit this eminent scientist during my first year of graduate school. How exciting and what an honour it was to be considered for this opportunity. It was an ‘arranged marriage’ for the next 4 years or so. I realized that working for this person would be difficult, but many of his trainees were very successful. I was therefore willing to deal with the challenges of working in his lab to reach my goal of becoming an independent scientist. However, when it was time to finalize plans to join his lab, he withdrew his offer as I needed my own funding to support my work. This expectation caught me by surprise as it was never discussed during the 4-plus years of my graduate studies. It truly was a disheartening experience. Nevertheless, the Steins were understanding and reassuring, and told me to take some time to read the latest *Cell*, *Science*, and *Nature* journals to find a research topic that I am most passionate about. They said that the subject you study as a fellow is usually what you continue to focus on for the rest of your career. That is when I came across Arnie’s study showing p53 is a tumour suppressor that blocks cancer (Finlay et al., 1989). I was fascinated by this concept and needed to learn how p53 functions in this capacity. I applied to Arnie’s lab and was fortunate to be accepted into his group. Agreeing to join Arnie’s lab was the easiest career decision that I have made. He is an exceptional scientist, a leader in cancer biology, and a fantastic mentor. His students and fellows were productive, successful, and enjoyed their work. The bottom line is that you should find a lab that is working in an exciting area, producing high quality data with strong publications and funding, and provides a nurturing, fun environment. Arnie’s group fulfilled all these criteria for me. Arnie was also willing to support me during my postdoctoral studies and he helped guide me through the process of applying for external funding, which resulted in fellowship offers from National Institutes of Health, American Cancer Society, and the Damon Runyon Cancer Research Foundation. The only downside in joining Arnie’s lab was space. There were >30 scientists in his group at that time: mostly postdoctoral fellows, a few students, two technicians (Angie Teresky and Jodi Martinez), several sabbatical faculty, and a wonderful administrative assistant (Kate James). He said that I could come to Princeton right away, but my bench would also be my desk (equivalent to one drawer length). I would also have access to the drawer below and a shelf above the bench, also equal to the length of the drawer. My alternative was to wait 6–8 months for a full bench and desk to become available. This was another easy decision, I joined the group immediately. It is amazing what you can accomplish with a 3′ × 3′ bench, drawer, and shelf!

**Figure 1:**
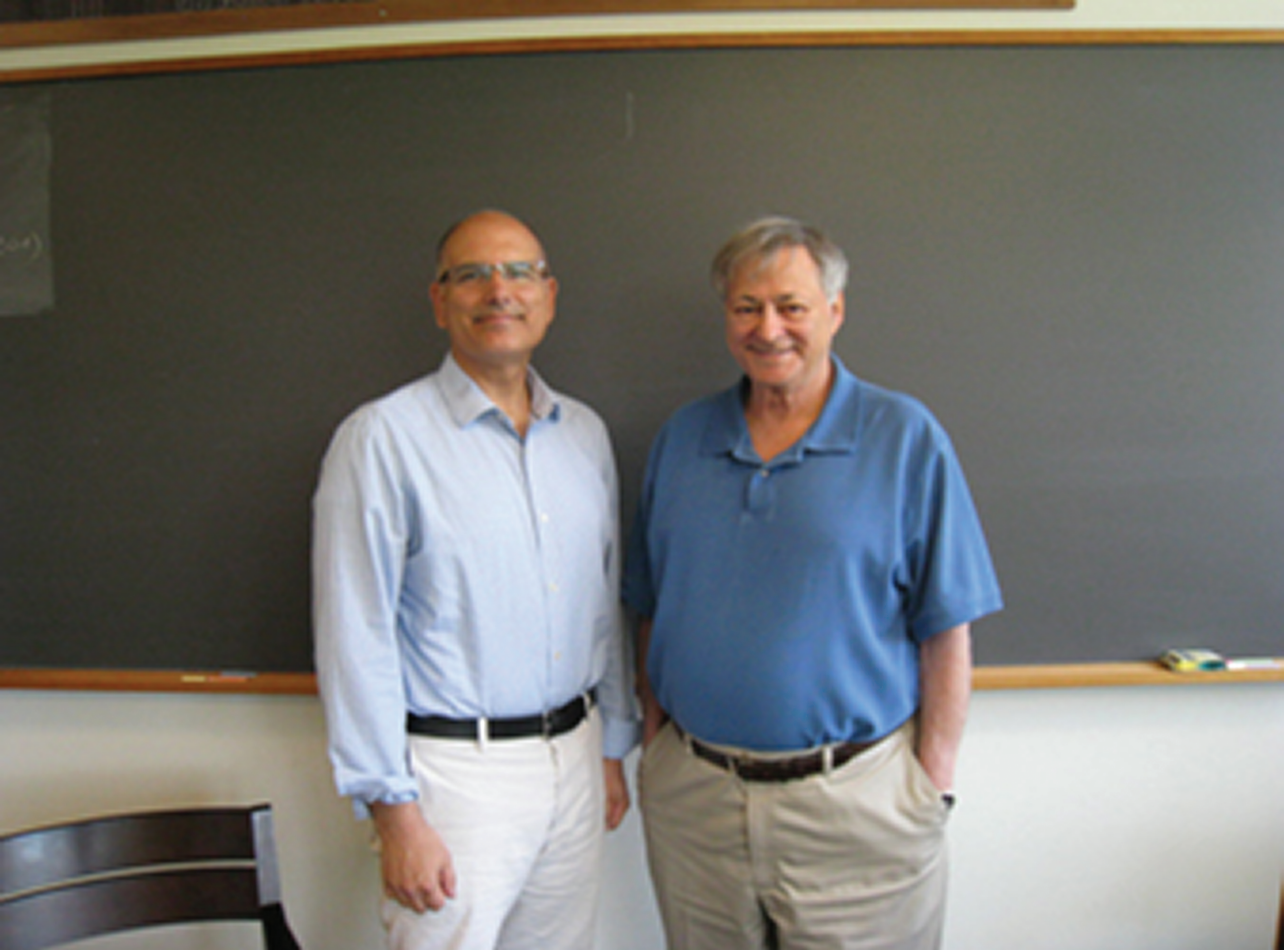
Jamil Momand and Arnie Levine (July 2017).

## Identification of Mdm2 as a p53 negative regulator

**Figure 2:**
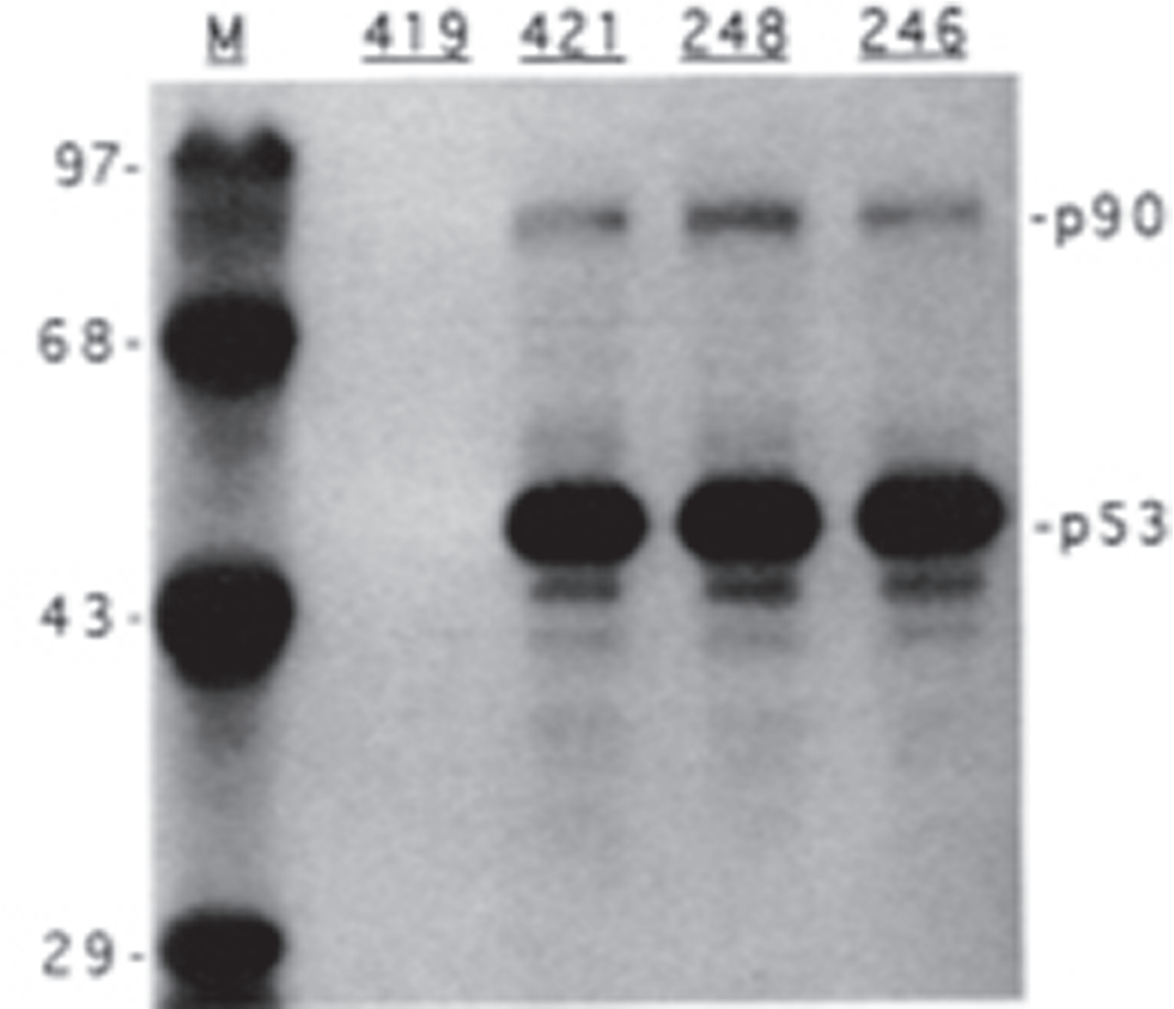
Autoradiogram of p53 immunoprecipitation from metabolically labelled cells (copied from Momand et al., 1992).

During the early 1990s, Arnie was travelling extensively and was often away from the office. However, one advantage of working in his lab was the relatively large number of postdoctoral fellows who came from diverse academic backgrounds, each with different skill sets. The fellows served as outstanding resources for biological and technical information and were always eager to help each other. As a new member of the group, I was interested in learning how to metabolically label cells and track p53 protein expression by immunoprecipitation-based approaches. Jamil Momand, a talented biochemist from the University of California at Los Angeles, was a first-year fellow in Arnie’s lab (Figure 1). He agreed for me to shadow him as he used these techniques to monitor the expression of a temperature-sensitive mouse mutant p53 (V135A) in transformed rat embryo fibroblasts (A1–5 cell line). The experiment took about one week and the results were clear. There was a strong p53 signal in each of the samples immunoprecipitated using a panel of monoclonal antibodies specific for different epitopes on p53, but not when using the PAb419 SV40 T-antigen-specific antibody (see Figure 2 as an example; copied from Momand et al., 1992). I distinctly recall Jamil questioning the identity of the ~90-kD protein. On a more simplistic level, I was just impressed with how well the technique had worked. This result sent Jamil on a mission to purify the 90-kD protein. A1 cells were grown to near confluence in ~500 tissue culture plates (15 cm each, consider the space and effort demands) and then shifted to 32°C to favour the wild-type p53 conformation. Lysates were prepared and passed over a PAb421 immunoaffinity column. The column was washed and the p53–p90 complex eluted using the PAb421 epitope peptide. The proteins were concentrated and separated on denaturing polyacrylamide gels. The p90 bands were excised and further concentrated using a customized polyacrylamide apparatus. The protein was electroblotted onto nitrocellulose and sent to William Lane at the Harvard Microchemistry Facility for Edman degradation amino terminal sequencing analysis. Three peptide sequences were returned, which matched the newly deposited murine Mdm2 sequence (GenBank) identified by Donna George at the University of Pennsylvania.

**Figure 3:**
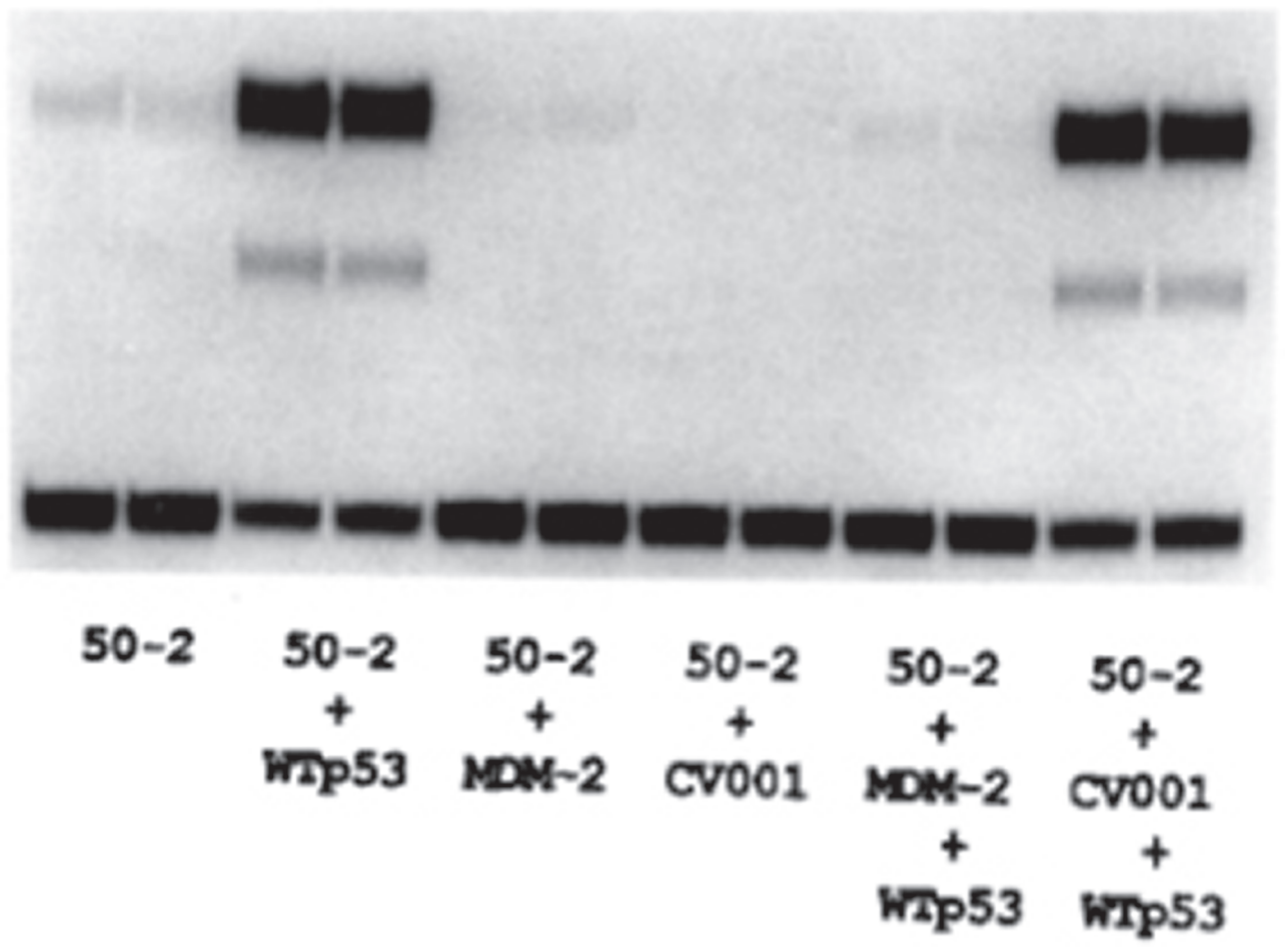
Promoter–reporter assay. Wild-type p53 strongly activates promoter containing p53 consensus sites (p50-2CAT) (lanes 3, 4 vs. 1, 2). Inclusion of Mdm-2 efficiently blocks p53 transactivation (lanes 9, 10 vs. 3, 4) (copied from Momand et al., 1992).

Donna George previously isolated a murine NIH3T3 fibroblast subclone that was tumourigenic, whereas the parental 3T3 cells were not (Cahilly-Snyder et al., 1987). A distinguishing feature of the tumourigenic 3T3 DM cells is the presence of multiple copies of double minutes, each harbouring three independent genes, i.e murine double minute-1 (Mdm-1), Mdm-2, and Mdm-3. Double minutes are extrachromosomal circular DNAs that are packaged into chromatin, but lack centromeres and telomeres. Double minutes are usually selected for their positive growth and survival advantages contributed by the amplification and overexpression of an oncogene(s). Stable overexpression of Mdm-2, but not Mdm-1 or Mdm-3, in NIH3T3 cells conferred oncogenic potential resulting in enhanced tumour growth (Fakharzadeh et al., 1991). Donna George was the first to recognize *Mdm-2* as an oncogene that functions in cellular growth control. Jamil’s discovery that Mdm-2 was a *bona fide* p53 binding protein provided the insight into how Mdm-2 may do so. If Mdm-2 is an oncoprotein, perhaps it functions by antagonizing p53 tumour suppressor activities. At that time, my project was focused on how p53 binds to DNA and regulates transcription. More specifically, I developed a minimal promoter–reporter assay using p53 DNA binding consensus sites from the muscle creatine kinase promoter (Zambetti et al., 1992). The reporter containing the binding sites is robustly induced in response to wild-type p53. Inclusion of Mdm-2 in this assay efficiently blocked p53 transactivation (Figure 3), which was the first demonstration that Mdm-2 is a negative regulator of the p53 tumour suppressor. This discovery set off a flurry of follow-up studies and Mdm-2 was quickly shown to be amplified and overexpressed in human sarcomas (Oliner et al., 1992). Structural studies revealed how Mdm-2 binds to p53 (N-terminus of p53 fits in hydrophobic cleft within the N-terminus of Mdm-2) (Kussie et al., 1996), which opened the door for developing drugs that could compete for binding and release p53 from Mdm-2, such as Nutlin (Vassilev et al., 2004). Even a family member was uncovered, Mdm-X/Mdm-4, which also represses p53 and has clinical significance in human cancer (e.g. retinoblastoma) (Shvarts et al., 1996; Laurie et al., 2006). There are now close to 10000 published studies on Mdm-2, international workshops focused on Mdm-2 (~every 2 years; Lu, 2017), and Mdm-2 inhibitors that are in clinical trials. Arnie’s mentorship and support provided the environment and opportunity to identify Mdm-2 as the 90-kD protein that negatively regulates p53. It really was an exciting wave to ride!

**Figure 4:**
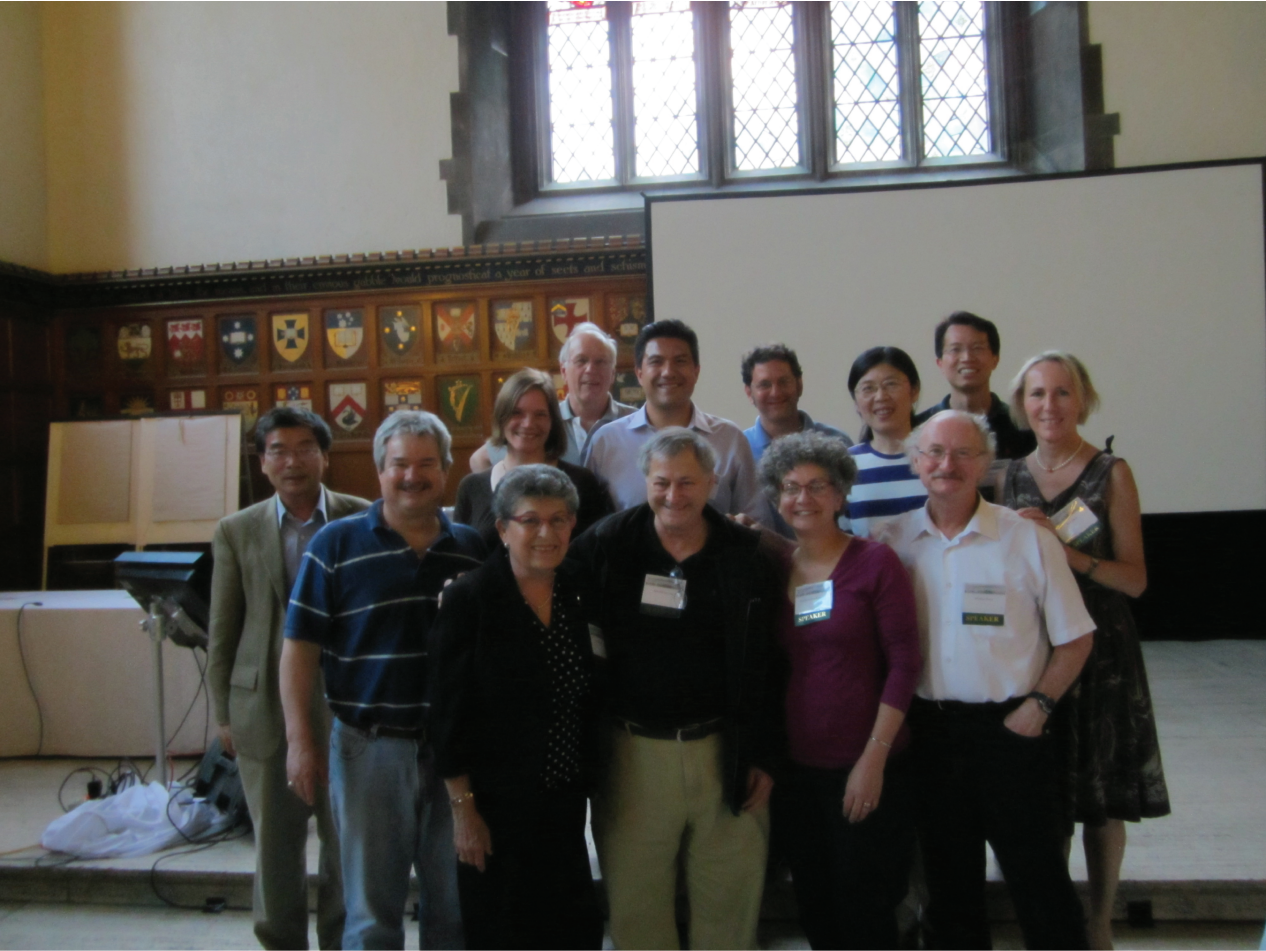
Arnie Levine’s ‘scientific family’ (partially represented) at 6th International Mutant p53 Workshop in Toronto (June 2013). Front row: Gerry Zambetti, Varda Rotter, Arnie Levine, Gigi Lozano, Moshe Oren; middle row: Hua Lu, Elke Markert, Darren Carpizo, Xin Yu, Ute Moll; back row: Alexel Vazquez (middle), Chang Chan (right).

## The Levine scientific family

Arnie has mentored many outstanding scientists such as Arthur Levinson (Genentech CEO and Chairman), Rudolf Jaenisch (National Academy of Sciences, National Academy of Medicine), Moshe Oren (Dean of the Faculty of Biology, Weizmann Institute of Science), and Gigi Lozano (Chair of Genetics, MD Anderson Cancer Center; National Academy of Sciences, National Academy of Medicine) to name just a few (Figure 4). He was also instrumental in building a state-of-the-art molecular research building on the campus of Princeton University (named after the renowned physician scientist Lewis Thomas), which housed a world-class faculty including Shirley Tilghman (HHMI; National Academy of Sciences; President of Princeton, 2001–2013), Tom Shenk (HHMI; National Academy of Sciences; Past President of American Society of Microbiology), Eric Wieschaus (Noble Prize in Physiology, 1995), and James Rothman (Nobel Prize in Physiology, 2013). It was a stimulating research environment that fostered outstanding academic achievements.

## Concluding remarks

I feel truly blessed to have had my path lead me to Arnie’s lab at Princeton. My postdoctoral experience was productive and enjoyable, and it was a privilege to have worked with such valued colleagues. I am grateful to have had such a wonderful mentor. We continue to stay in touch and Arnie still provides invaluable scientific and career development advice. He has visited St. Jude on multiple occasions and just last year presented a lecture on cancer biology to our inaugural class of St. Jude Graduate Students. It was not surprising that they voted him the best lecturer. Arnie, special wishes on your 80th birthday.
